# Trophectoderm-Specific Knockdown of LIN28 Decreases Expression of Genes Necessary for Cell Proliferation and Reduces Elongation of Sheep Conceptus

**DOI:** 10.3390/ijms21072549

**Published:** 2020-04-06

**Authors:** Asghar Ali, Mark D. Stenglein, Thomas E. Spencer, Gerrit J. Bouma, Russell V. Anthony, Quinton A. Winger

**Affiliations:** 1 Department of Biomedical Sciences, Animal Reproduction and Biotechnology Laboratory, 1683 Campus Delivery, Colorado State University, Fort Collins, CO 80523, USA; asghar.ali@colostate.edu (A.A.); gerrit.bouma@colostate.edu (G.J.B.); Russ.Anthony@ColoState.edu (R.V.A.); 2 Department of Microbiology, Immunology and Pathology, Colorado State University, Fort Collins, CO 80523, USA; mark.stenglein@colostate.edu; 3 Animal Science Research Center, College of Agriculture, Food and Natural Resources, University of Missouri, Columbia, MO 65211, USA; spencerte@missouri.edu

**Keywords:** trophectoderm, placenta, cell proliferation, *let-7* miRNAs, gene regulation

## Abstract

LIN28 inhibits *let-7* miRNA maturation which prevents cell differentiation and promotes proliferation. We hypothesized that the LIN28-*let-7* axis regulates proliferation-associated genes in sheep trophectoderm in vivo. Day 9-hatched sheep blastocysts were incubated with lentiviral particles to deliver shRNA targeting LIN28 specifically to trophectoderm cells. At day 16, conceptus elongation was significantly reduced in LIN28A and LIN28B knockdowns. *Let-7* miRNAs were significantly increased and IGF2BP1-3, HMGA1, ARID3B, and c-MYC were decreased in trophectoderm from knockdown conceptuses. Ovine trophoblast (OTR) cells derived from day 16 trophectoderm are a useful tool for in vitro experiments. Surprisingly, LIN28 was significantly reduced and *let-7* miRNAs increased after only a few passages of OTR cells, suggesting these passaged cells represent a more differentiated phenotype. To create an OTR cell line more similar to day 16 trophectoderm we overexpressed LIN28A and LIN28B, which significantly decreased *let-7* miRNAs and increased IGF2BP1-3, HMGA1, ARID3B, and c-MYC compared to control. This is the first study showing the role of the LIN28-*let-7* axis in trophoblast proliferation and conceptus elongation in vivo. These results suggest that reduced LIN28 during early placental development can lead to reduced trophoblast proliferation and sheep conceptus elongation at a critical period for successful establishment of pregnancy.

## 1. Introduction

Early placental development is one of the main factors determining perinatal fetal growth and postnatal fetal and maternal health. In humans, blastocyst implantation is an invasive process that occurs 7–9 days after fertilization [1]. Rapidly proliferating cytotrophoblast cells (CTBs) are the progenitor trophoblast cells which proliferate as well as differentiate into different trophoblast lineages throughout gestation [2]. If the balance between proliferation and differentiation of CTBs is dysregulated, it can result in severe disorders including preterm birth, intrauterine growth restriction (IUGR), and preeclampsia [3,4]. These pregnancy related disorders affect about a third of human pregnancies [5].

In sheep, the blastocyst hatches out of the zona pellucida at day 8–9 and is surrounded by a single layer of mononuclear cells called trophectoderm (TE) [6]. Instead of invading the uterus, the hatched blastocyst elongates from day 11–16 due to rapid proliferation of trophoblast cells and adopts a filamentous shape comprised of mainly extraembryonic trophoblast cells [7,8,9]. Conceptus elongation is critical for implantation, placentation, and successful establishment of pregnancy in sheep [10,11,12]. Reduced conceptus elongation and compromised placental function in domestic ruminants is one of the main causes of embryonic mortality resulting in reduced fertility [13,14,15]. Rapid trophoblast proliferation is an important phenomenon during early stages of pregnancy in both humans and domestic ruminants. The molecular mechanisms involved in regulating trophoblast proliferation and invasion are not well understood. Therefore, exploring the genes involved in sheep trophectoderm elongation can help to better understand the reasons for reduced fertility in domestic ruminants and to improve the diagnosis and treatment of various pregnancy-related disorders in humans.

Trophoblast proliferation and differentiation is an intensively regulated process, and the role of several genes in placental development has been studied using various in vivo and in vitro models [16,17,18,19,20]. The pluripotency factor LIN28 is a highly conserved RNA binding protein which is expressed in placenta and has two paralogs, LIN28A and LIN28B [21,22]. It is usually described as a protooncogene due to its ability to regulate and stabilize oncogenes at the post-transcriptional level in tumor cells [23,24]. It also inhibits the biogenesis of lethal-7 (*let-7)* miRNAs in mammalian cells by binding pri-*let-7* and pre*-let-7* [25,26,27,28,29,30]. LIN28 is low and *let-7* miRNAs are high in differentiated cells and adult tissues, hence *let-7* miRNAs are considered markers of cell differentiation [31,32,33]. *Let-7* miRNAs reduce the expression of different proliferation factors either by directly targeting their mRNA or through chromatin-dependent pathways by targeting the ARID3B-complex, which is comprised of AT-Rich Interaction Domain 3A (ARID3A), AT-Rich Interaction Domain 3B (ARID3B) and lysine demethylase 4C (KDM4C) [18,34]. We recently showed that term human placentas from IUGR pregnancies had reduced LIN28A and LIN28B and high *let-7* miRNAs compared to term human placentas from control pregnancies [18]. We further demonstrated that LIN28B is localized to cytotrophoblast cells in human placenta, and knockout of LIN28 in immortalized first trimester human trophoblast (ACH-3P) cells leads to an increase in *let-7* miRNAs, reduced expression of proliferation-associated genes, and reduced cell proliferation [18,19,20].

Insulin like growth factor 2 mRNA binding proteins (*IGF2BP1, IGF2BP2, IGF2BP3*), high mobility group AT-hook 1 (*HMGA1*), *ARID3B,* and MYC protooncogene (*c-MYC)* are all *let-7* miRNA targets with known roles in cell proliferation [18,35,36,37,38,39,40,41]. IGF2BPs are highly conserved RNA binding oncofetal proteins with three paralogs, IGF2BP1, IGF2BP2, and IGF2BP3 [42]. By binding different mRNAs, IGF2BPs decide the fate of those mRNAs by controlling their localization, stability, and translation [40]. Many studies have reported the role of IGF2BPs in cell proliferation, cell invasion, tumorigenesis, and embryogenesis [40,41,42,43,44,45,46,47,48,49,50,51]. IGF2BPs have also been found in sheep trophoblast cells suggesting their role in rapid proliferation of these cells [52]. HMGA1 promotes invasion of trophoblast cells and reduced levels of HMGA1 has been linked to pathogenesis of preeclampsia [53,54]. ARID3B binds with ARID3A and KDM4C to form the ARID3B-complex. The ARID3B-complex plays a vital role in cell proliferation by transcriptional regulation of stemness genes including *HMGA1*, *c-MYC*, vascular endothelial growth factor A (*VEGF-A*), and Wnt family member 1 (*WNT1*) [18,34,55,56,57,58,59]. ARID3B knockout in immortalized first trimester human trophoblast cells results in reduced proliferation of these cells [18]. The *c-MYC* protooncogene has been identified as a proliferation factor in human cytotrophoblast cells and its level is reduced when cytotrophoblasts differentiate into syncytiotrophoblast [60].

To date, the role of LIN28-*let-7* miRNA axis in trophoblast cells has not been studied in vivo. The aim of this study was to demonstrate the role of LIN28-*let-7* axis in the regulation of proliferation-associated genes in trophoblast cells in vivo. We used sheep as an in vivo model to generate trophectoderm specific knockdown of LIN28A or LIN28B by infecting day 9-hatched blastocysts with shRNA-expressing lentiviral particles. This way, only the trophoblast cells will be infected by the lentiviral particles [61,62,63] and any phenotype will be due to knockdown of LIN28A or LIN28B in trophoblast cells. We hypothesized that the LIN28-*let-7* miRNAs axis plays an important role in sheep trophoblast cell proliferation and conceptus elongation by regulating the expression of genes associated with cell proliferation including *IGF2BP1, IGF2BP2, IGF2BP3*, *HMGA1*, *ARID3B,* and *c-MYC*.

## 2. Results

### 2.1. LIN28 Knockdown in Trophectoderm Resulted in Reduced Proliferation of Trophoblast Cells and Lower Expression of Proliferation-Associated Genes

**Figure 1:**
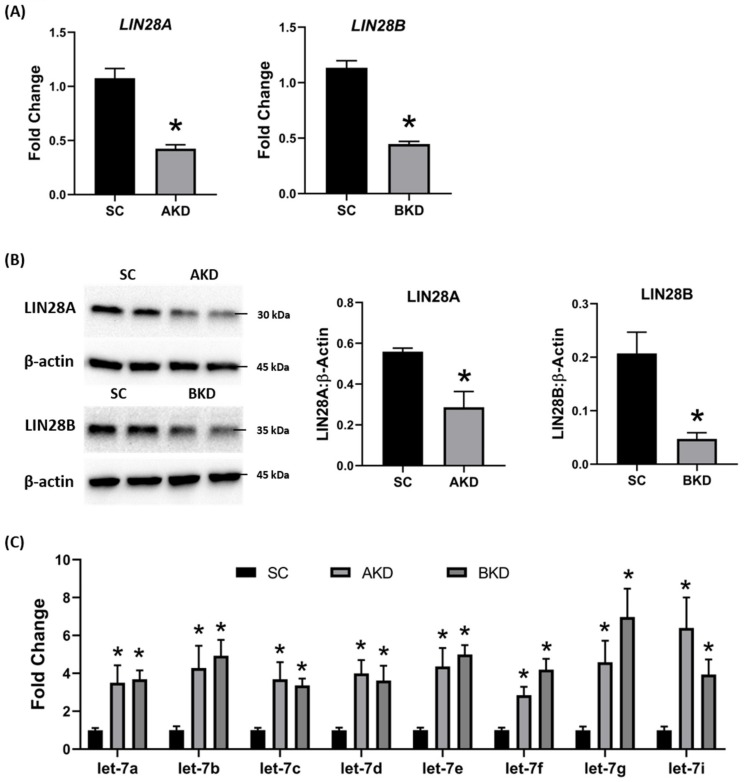
LIN28A or LIN28B knockdown and *let-7* miRNAs in day 16 sheep TE. (**A**) *LIN28A* and *LIN28B* mRNA in AKD (*n* = 5) and BKD (*n* = 6) day 16 TE compared to SC (*n* = 6). (**B**) Representative immunoblots for LIN28A, LIN28B, and β-actin in AKD, BKD, and SC day 16 TE, and densitometric analysis. (**C**) *Let-7* miRNAs in AKD and BKD day 16 TE and SC. * *p* < 0.05 vs. SC.

Day 9-hatched blastocysts were infected with lentiviral particles expressing shRNA to knockdown LIN28A (AKD) or LIN28B (BKD), or scramble control shRNA (SC). The blastocysts treated with lentiviral particles were surgically transferred to synchronized ewes at day 9 of estrus. The conceptuses were collected at day 16 of gestation and trophectoderm (TE) was separated from embryo. LIN28A and LIN28B mRNAs and proteins were quantified by real-time PCR and Western blot. LIN28A mRNA and protein was significantly reduced in AKD TE while LIN28B mRNA and protein was significantly reduced in BKD TE compared to SC (Figure 1A,B). As expected, due to reduced LIN28, *let-7* miRNAs (*let-7a, let-7b, let-7c, let-7d, let-7e, let-7f, let-7g, let-7i*) were significantly higher in AKD and BKD TE compared to SC, and there was no significant change in *let-7* miRNAs between AKD and BKD TE (Figure 1C). These results suggest that reduced LIN28A or LIN28B led to significant increase in *let-7* miRNAs.

**Figure 2:**
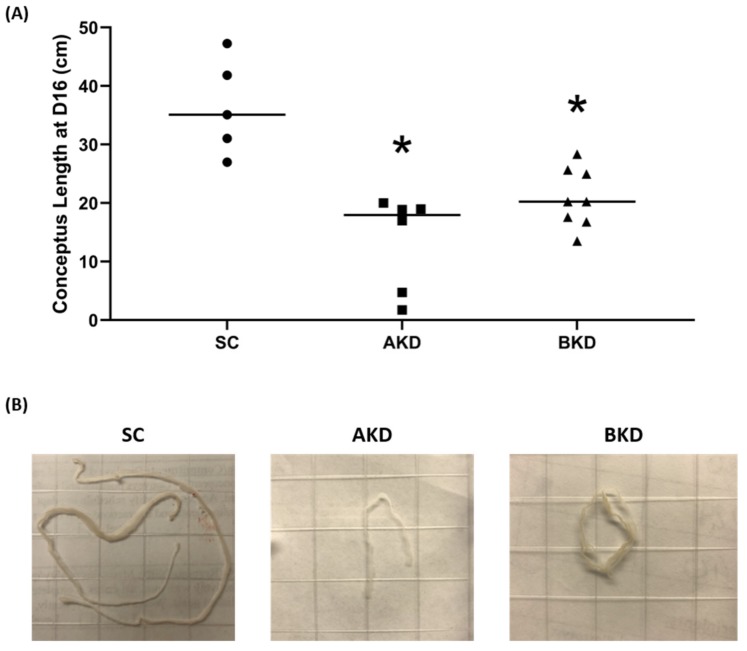
(**A**) Conceptus length at day 16 after LIN28A knockdown (AKD, *n* = 6) and LIN28B knockdown (BKD, *n* = 8) compared to scramble control (SC, *n* = 5). (**B**) Representative images of day 16 sheep conceptuses for AKD, BKD, and SC day 16 TE; * *p* < 0.05 vs. SC.

To determine the effect of LIN28 knockdown on conceptus elongation, we measured the length of TE. Knockdown of LIN28A or LIN28B resulted in a significant reduction in day 16 TE length compared to SC (Figure 2A,B). There was no significant difference in elongation of AKD vs. BKD TE. This data suggest knockdown of either LIN28A or LIN28B in vivo resulted in reduced proliferation of trophoblast cells.

**Figure 3:**
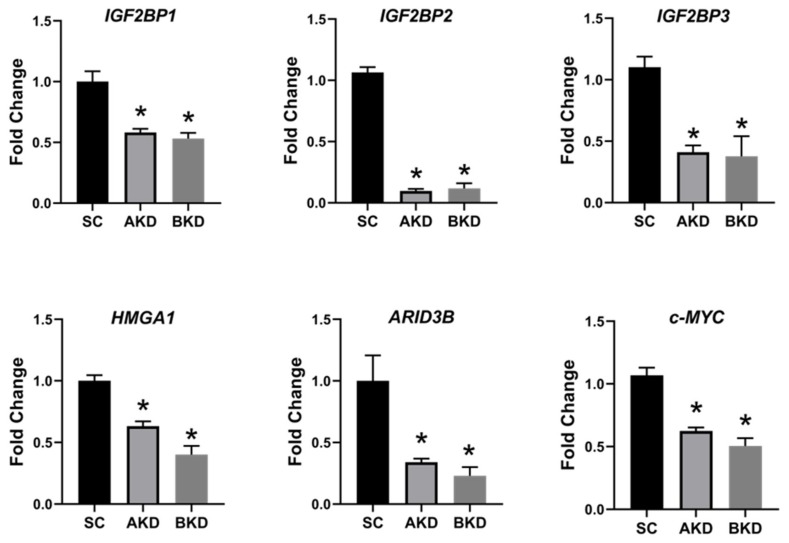
*IGF2BP1, IGF2BP2, IGF2BP3, HMGA1, ARID3B,* and *c-MYC* mRNA in AKD and BKD day 16 TE compared to SC (*n* = 5), * *p* < 0.05 vs. SC.

**Figure 4:**
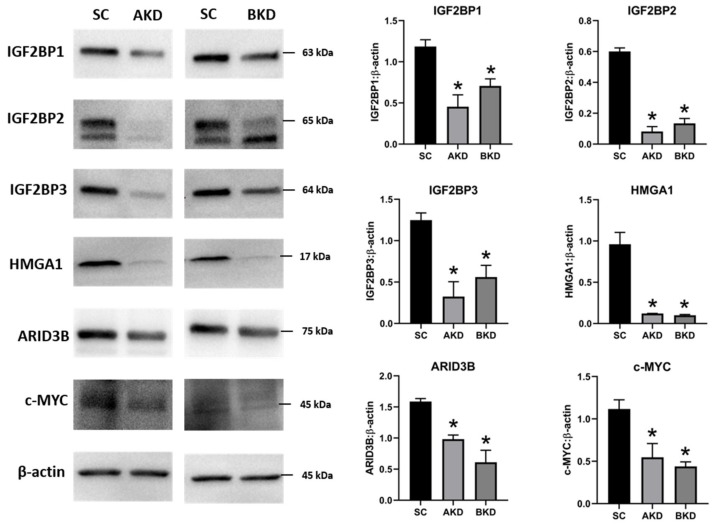
Representative immunoblots for IGF2BP1, IGF2BP2, IGF2BP3, HMGA1, ARID3B, c-MYC, and β-actin, and densitometric analysis of immunoblotting results in AKD and BKD day 16 sheep TE compared to SC (*n* = 3), * *p* < 0.05 vs. SC.

Due to the potential for reduced proliferation of trophoblast cells in the AKD and BKD conceptuses, we measured the mRNA and protein levels of *let-7*-regulated proliferation-associated genes. The mRNA and protein levels of IGF2BP1, IGF2BP2, IGF2BP3, HMGA1, ARID3B, and c-MYC were significantly reduced in AKD and BKD day 16 TE compared to SC (Figure 3, Figure 4). These results suggest that high *let-7* miRNAs in AKD and BKD TE led to a significant reduction in expression of IGF2BP1, IGF2BP2, IGF2BP3, HMGA1, ARID3B, and c-MYC, and the reduced proliferation of trophoblast cells in AKD and BKD TE is due to significantly reduced expression of these proliferation-associated genes.

### 2.2. Ovine Trophoblast Cells Generated from Day 16 Trophectoderm Had a Significant Reduction in LIN28

**Figure 5:**
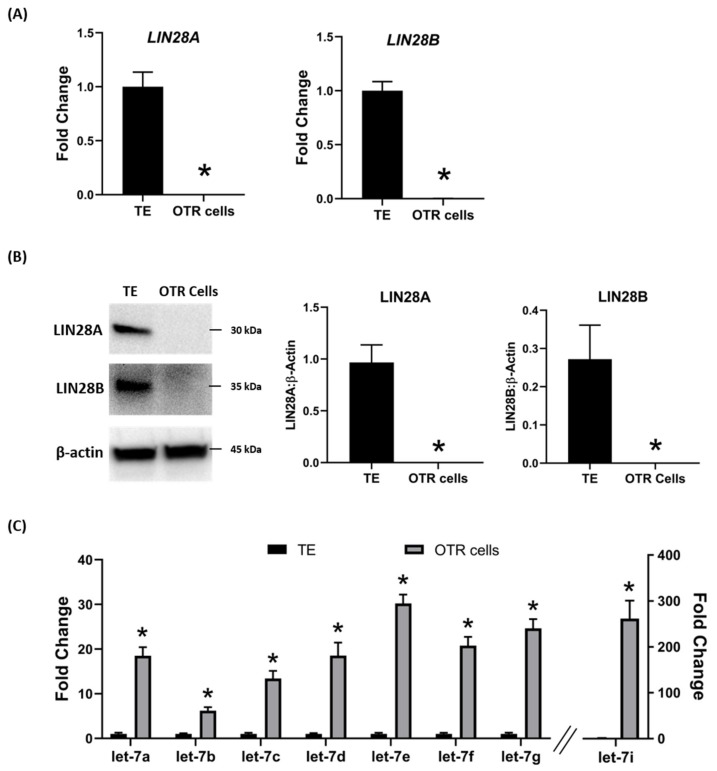
LIN28A, LIN28B, and *let-7* miRNAs in non-immortalized ovine trophoblast (OTR) cells. (**A**) *LIN28A* and *LIN28B* mRNA in OTR cells compared to day 16 sheep TE. (**B**) Representative immunoblots for LIN28A, LIN28B, and β-actin, and densitometric analysis of immunoblotting results in OTR cells compared to day 16 TE. (**C**) *Let-7* miRNAs in OTR cells compared to day 16 TE (*n* = 3), * *p* < 0.05 vs. TE.

To further investigate the regulation of ovine trophoblast cell proliferation by LIN28 in vitro, we used day 16 TE to generate ovine trophoblast cells. The day 16 TE was minced and plated in collagen-coated plates and was passaged to obtain a cell line. The cells used for further experiments were collected at passage 4–6, so we called these cells non-immortalized ovine trophoblast (OTR) cells. Interestingly, real-time PCR data showed that OTR cells had a significant reduction in *LIN28A* and *LIN28B* mRNAs compared to day 16 TE (Figure 5A). Densitometric analysis of Western blots showed that LIN28A and LIN28B proteins were also significantly reduced in OTR cells compared to day 16 TE (Figure 5B). Furthermore, real-time PCR data showed significant increase in *let-7* miRNAs (*let-7a, let-7b, let-7c, let-7d, let-7e, let-7f, let-7g, let-7i*) in OTR cells compared to day 16 TE (Figure 5C). The significantly reduced LIN28 and high *let-7* miRNAs in OTR cells after only 4–6 passages suggest that these cells differentiated to a different phenotype compared to trophoblast cells in day 16 TE.

**Figure 6:**
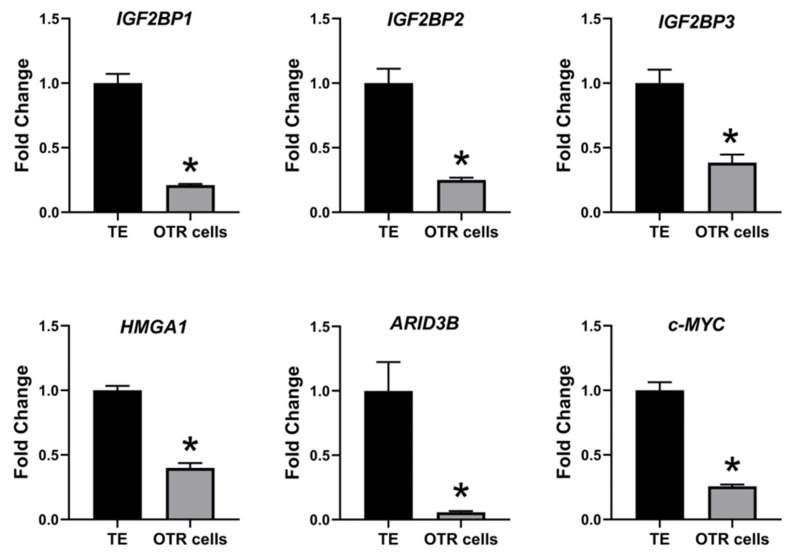
*IGF2BP1, IGF2BP2, IGF2BP3, HMGA1, ARID3B,* and *c-MYC* mRNA in OTR cells compared to day 16 TE (*n* = 3), * *p* < 0.05 vs. TE.

**Figure 7:**
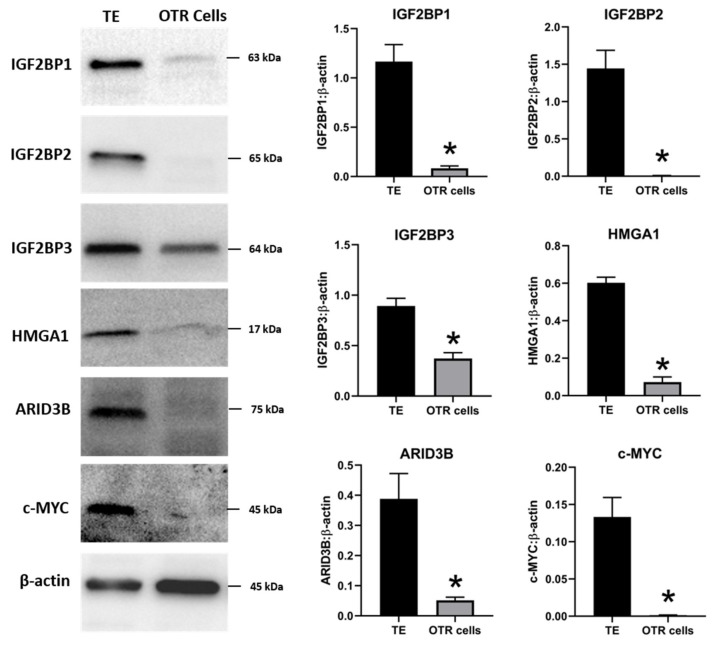
Representative immunoblots for IGF2BP1, IGF2BP2, IGF2BP3, HMGA1, ARID3B, c-MYC, and β-actin, and densitometric analysis of immunoblotting results in OTR cells compared to day 16 TE (*n* = 3), * *p* < 0.05 vs. TE.

The effect of low LIN28 and high *let-7* miRNAs on proliferation-associated genes in OTR cells was determined by measuring IGF2BP1, IGF2BP2, IGF2BP3, HMGA1, ARID3B, and c-MYC mRNAs and proteins. Real-time PCR showed that mRNA levels of *IGF2BP1, IGF2BP2, IGF2BP3, HMGA1, ARID3B,* and *c-MYC* were significantly reduced in OTR cells compared to day 16 TE (Figure 6). Densitometric analysis of Western blots revealed a significant reduction in protein levels of IGF2BP1, IGF2BP2, IGF2BP3, HMGA1, ARID3B, and c-MYC in OTR cells compared to day 16 TE (Figure 7). These results suggest that reduced LIN28 and high *let-7* miRNAs led to reduced expression of proliferation-associated genes in OTR cells.

The OTR cells originated from day 16 TE undergo senescence after only a few passages. Therefore, the OTR cells were immortalized by overexpressing human telomerase reverse transcriptase (hTERT) to keep them growing for further in vitro experiments. The newly generated immortalized cells were referred to as immortalized ovine trophoblast (iOTR) cells.

### 2.3. Overexpression of LIN28 in iOTR Cells Resulted in Increased Expression of Proliferation-Associated Genes

**Figure 8:**
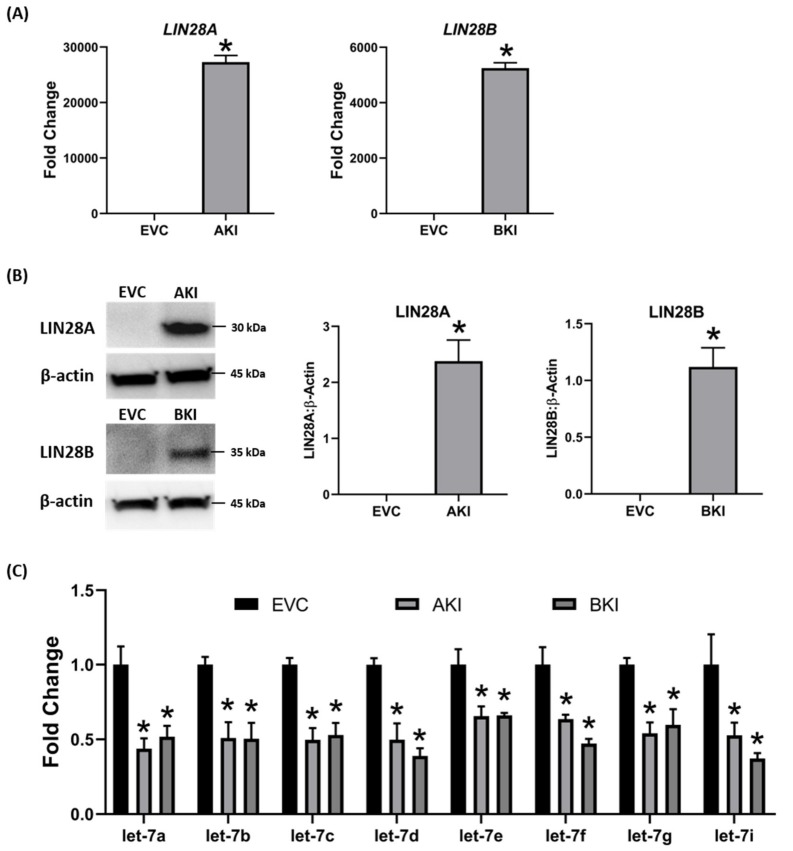
LIN28A, LIN28B, and *let-7* miRNAs in LIN28A knockin (AKI) and LIN28B knockin (BKI) immortalized ovine trophoblast (iOTR) cells. (**A**) *LIN28A* and *LIN28B* mRNA in AKI and BKI iOTR cells compared to expression vector control (EVC). (**B**) Representative immunoblots for LIN28A, LIN28B, and β-actin, and densitometric analysis of immunoblotting results in AKI and BKI iOTR cells compared to EVC. (**C**) *Let-7* miRNAs in AKI and BKI iOTR cells compared to EVC (*n* = 3), * *p* < 0.05 vs. EVC.

To determine if LIN28 overexpression will rescue the expression of proliferation-associated genes, the iOTR cells were infected with lentiviral particles to generate LIN28A knockin (AKI) or LIN28B knockin (BKI), or lentiviral particles with empty expression vector as expression vector control (EVC). Real-time PCR data showed that AKI iOTR cells had a significant increase in *LIN28A* mRNA while BKI iOTR cells had a significant increase in *LIN28B* mRNA compared to EVC (Figure 8A). The densitometric analysis of Western blots showed a significant increase in LIN28A protein in AKI and significant increase in LIN28B protein in BKI iOTR cells compared to EVC (Figure 8B). Moreover, the real-time PCR data showed that *let-7* miRNAs (*let-7a, let-7b, let-7c, let-7d, let-7e, let-7f, let-7g, let-7i*) were significantly reduced in both AKI and BKI iOTR cells compared to EVC (Figure 8C). These results suggest that increased expression of either LIN28A or LIN28B leads to reduction in *let-7* miRNAs.

**Figure 9:**
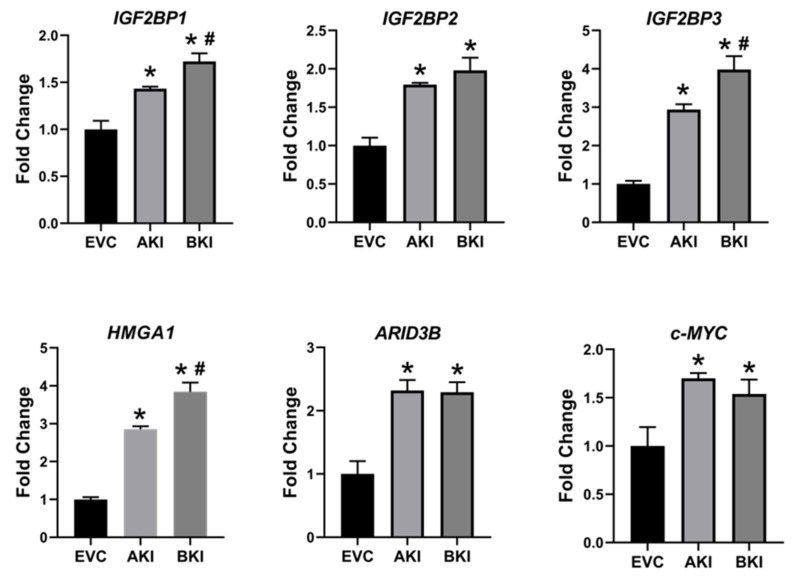
*IGF2BP1, IGF2BP2, IGF2BP3, HMGA1, ARID3B,* and *c-MYC* mRNA in AKI and BKI iOTR cells compared to EVC (*n* = 3), where * *p* < 0.05 vs. EVC and # *p* < 0.05 vs AKI.

**Figure 10:**
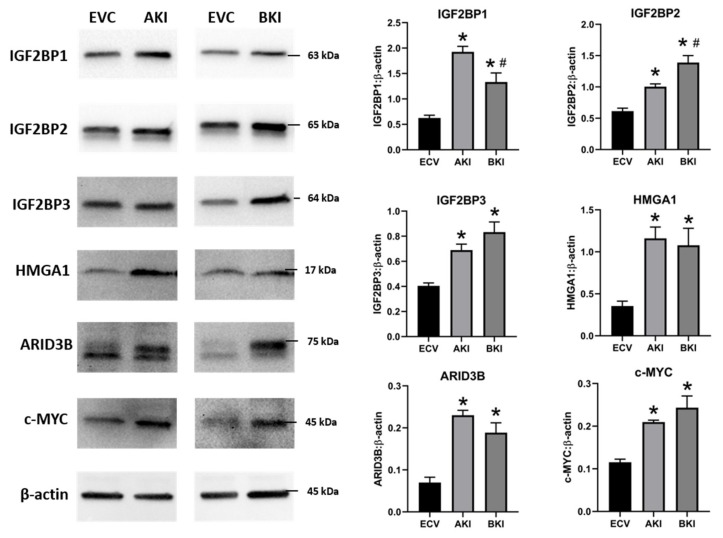
Representative immunoblots for IGF2BP1, IGF2BP2, IGF2BP3, HMGA1, ARID3B, c-MYC, and β-actin, and densitometric analysis of immunoblotting results in AKI and BKI iOTR cells compared to EVC (*n* = 3), where * *p* < 0.05 vs. EVC and # *p* < 0.05 vs AKI.

To determine the effect of LIN28 overexpression and reduction in *let-7* miRNAs on expression of proliferation-associated genes, real-time PCR and Western blot analysis were done. Real-time PCR showed that mRNA levels of *IGF2BP1, IGF2BP2, IGF2BP3, HMGA1, ARID3B,* and *c-MYC* were significantly increased in both AKI and BKI iOTR cells compared to EVC (Figure 9). Densitometric analysis of Western blots showed significant increase in IGF2BP1, IGF2BP2, IGF2BP3, HMGA1, ARID3B, and c-MYC proteins in both AKI and BKI iOTR cells compared to EVC (Figure 10). These results suggest that the expression of proliferation-associated genes in immortalized ovine trophoblast cells is regulated by the LIN28-*let-7* axis.

### 2.4. Overexpression of LIN28 Led to Significant Increase in Trophoblast Cell Proliferation

**Figure 11:**
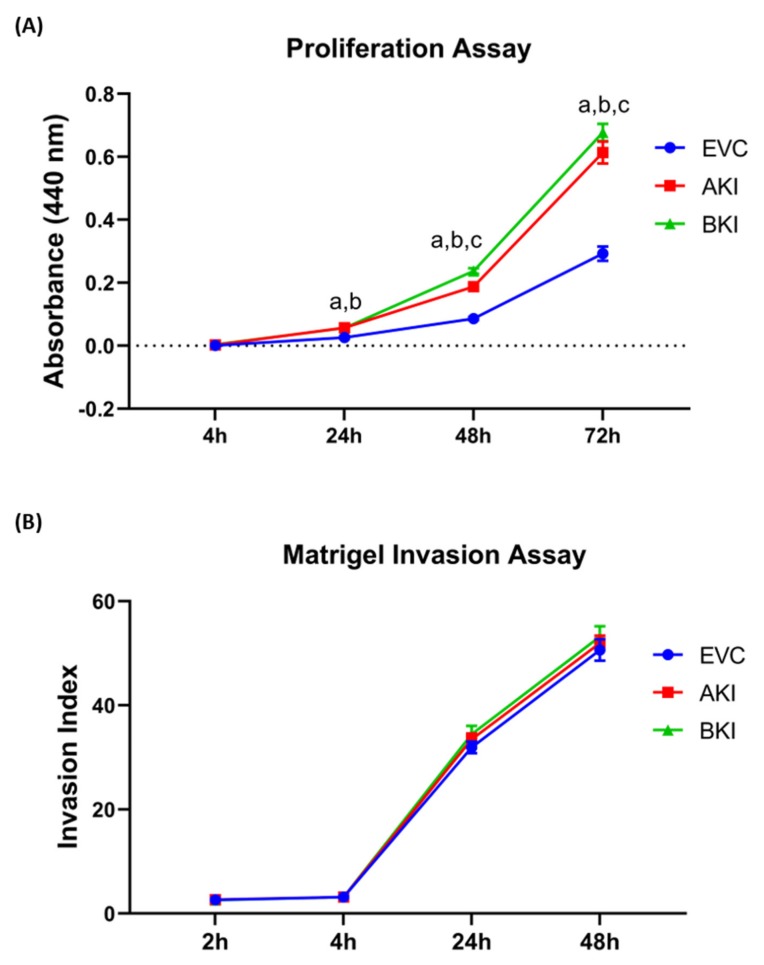
(**A**) Proliferation of AKI, BKI, and EVC iOTR cells (*n* = 4/treatment) was measured after 4 h, 24 h, 48 h, and 72 h using Quick Cell Proliferation Assay Kit. (**B**) Invasion of AKI, BKI, and EVC iOTR cells (*n* = 4) measured after 2 h, 4 h, 24 h, and 48 h using the Matrigel Invasion Assay Kit; where a, *p* < 0.05 for AKI vs. EVC; b, *p* < 0.05 for BKI vs. EVC; and c, *p* < 0.05 for BKI vs. AKI.

The role of LIN28-*let-7* miRNA axis on the functionality of iOTR cells was determined by measuring proliferation of AKI and BKI iOTR cells compared to EVC after 4 h, 24 h, 48 h, and 72 h. The results showed that proliferation of both AKI and BKI iOTR cells was significantly increased at 24 h, 48 h, and 72 h compared to EVC (Figure 11A). Furthermore, proliferation of BKI iOTR cells was not different at 24 h but was significantly higher at 48 h and 72 h compared to AKI iOTR cells (Figure 11A). The matrigel invasion assay showed that there was no significant change in the invasion index of AKI and BKI iOTR cells compared to EVC (Figure 11B). These results suggest that increased proliferation of AKI and BKI iOTR cells is due to increased expression of proliferation-associated genes in these cells compared to EVC.

## 3. Discussion

The pluripotency factors LIN28A and LIN28B inhibit the maturation of *let-7* miRNAs [39,62]. Recently, we showed that both Lin28A and LIN28B were significantly decreased and levels of *let-7* miRNAs (*let-7a, let-7b, let-7c, let-7d, let-7e, let-7f, let-7g, let-7i*) were significantly increased in term human placentas from IUGR pregnancies compared to control pregnancies [18]. We further demonstrated that double knockout of LIN28A and LIN28B in immortalized first trimester human trophoblast (ACH-3P) cells resulted in more robust increase in *let-7* miRNAs compared to knockout of either LIN28A or LIN28B. Similarly, double knockin of LIN28A and LIN28B in Sw.71 cells led to more robust decrease in *let-7* miRNAs compared to knockin of either LIN28A or LIN28B [18]. In this study, we show that RNA interference (RNAi) of LIN28A or LIN28B in sheep TE in vivo resulted in significant increase in *let-7* miRNAs (*let-7a, let-7b, let-7c, let-7d, let-7e, let-7f, let-7g, let-7i*). Moreover, the conceptus elongation was significantly reduced after RNAi of LIN28A or LIN28B in TE.

Although animal models with global gene knockout or knockdown have been extensively and successfully used in many studies, it is difficult to exclude the effect of global gene manipulation while focusing on mechanisms involved in one tissue type or organ. Incubating the day 9-hatched sheep blastocyst with shRNA expressing lentiviral particles caused viral infection of only trophoblast cells while all other cells including the inner cell mass were spared of lentiviral infection [61]. Hence, significant reduction in LIN28A or LIN28B in day 16 TE conceptus was restricted to the trophoblast cells only. Conceptus elongation in sheep was due to rapid proliferation of trophoblast cells [7,8,9]; therefore, a significant reduction in conceptus length at day 16 after trophectoderm-specific LIN28A or LIN28B knockdown indicates reduced proliferation of trophoblast cells.

**Figure 12:**
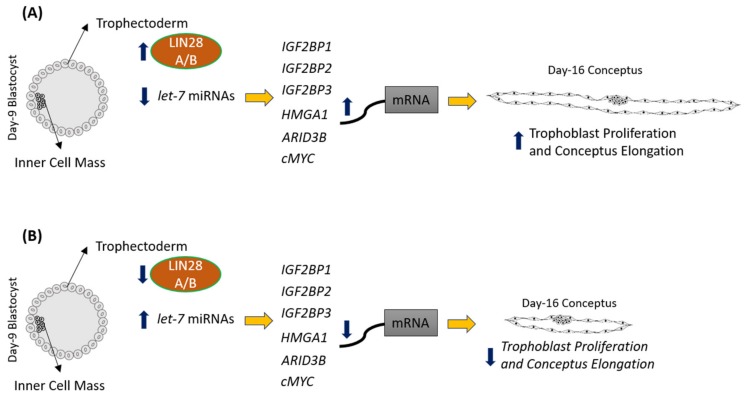
Graphical abstract. (**A**) In control day 9 TE, *let-7* miRNAs are low because of high LIN28A and LIN28B. Because of low levels of let-7 miRNAs, IGF2BP1, IGF2BP2, IGF2BP3, HMGA1, ARID3B, and c-MYC are higher leading to increased proliferation of trophoblast cells and hence conceptus elongation. (**B**) In LIN28A or LIN28B KD day 9 TE, *let-7* miRNAs are higher. Elevated let-7 miRNAs target *IGF2BP1, IGF2BP2, IGF2BP3, HMGA1, ARID3B,* and *c-MYC*, leading to reduced expression of these genes, leading to reduced proliferation of trophoblast cells and, hence, reduced conceptus elongation.

LIN28 is a part of a complex genetic pathway known to regulate multiple downstream targets [64]. Inhibition of biogenesis of mature *let-7* miRNAs is one of the main pathways through which LIN28 regulates the expression of its downstream targets [32,65]. *Let-7* miRNAs reduce expression of many genes by degrading their mRNAs or inhibiting translation [39]. Our results show that RNAi of LIN28A or LIN28B and resultant increase in *let-7* miRNAs in day 16 TE significantly reduced IGF2BP1, IGF2BP2, IGF2BP3, HMGA1, ARID3B, and c-MYC. Previous studies have shown the role of IGF2BP1, IGF2BP2, IGF2BP3, HMGA1, ARID3B, and c-MYC in cell proliferation [17,38,39,40,43,45,46,48,51], suggesting that reduced proliferation of trophoblast cells after LIN28A or LIN28B knockdown in TE is due to reduced expression of proliferation-associated genes (Figure 12).

To further investigate the role of the LIN28-*let-7* miRNA axis in sheep trophoblast cells in vitro, OTR cells were generated from day 16 TE. Surprisingly, both LIN28A and LIN28B were depleted and the *let-7* miRNAs were significantly higher in OTR cells compared to day 16 TE. The senescence of OTR cells after only a few passages along with high *let-7* miRNAs suggests that these cells are a differentiated phenotype of trophoblast cells compared to day 16 TE. Furthermore, the expression of proliferation-associated genes including IGF2BP1, IGF2BP2, IGF2BP3, HMGA1, ARID3B, and c-MYC was also significantly reduced in OTR cells compared to day 16 TE. To overcome the OTR cell senescence, we generated immortalized ovine trophoblast (iOTR) cells by expressing hTERT in these cells. The OTR cells were differentiated and depleted LIN28 and immortalization with hTERT expression did not change LIN28 expression in iOTR cells. Immortalized human first trimester trophoblast cells (Sw.71 cells) were also generated by expressing hTERT in first trimester human trophoblast cells at passage 3 [66]. Sw.71 cells also had depleted LIN28A and LIN28B and high *let-7* miRNAs compared to other trophoblast-derived cell lines such as ACH-3P cells [18]. The iOTR cells generated in this study were similar to Sw.71 cells in terms of LIN28 and *let-7* miRNAs expression, which may be because both cell lines were generated by immortalizing the passaged trophoblast cells. The contrasting levels of LIN28, *let-7* miRNAs, and *let-7* miRNA target genes between day 16 TE and iOTR cells should be taken in consideration if using these cells for further studies.

To generate iOTR cells that were more similar to day 16 TE, we overexpressed LIN28A or LIN28B in iOTR cells. Overexpression of LIN28A or LIN28B in iOTR cells led to a significant decrease in *let-7* miRNAs and significant increase in expression of IGF2BP1, IGF2BP2, IGF2BP3, HMGA1, ARID3B, and c-MYC compared to control. The proliferation of both AKI and BKI iOTR cells was significantly increased compared to control; however, there was no change in cell invasion. We recently showed that knockout of LIN28A and LIN28B in ACH-3P cells reduced cell proliferation but did not affect cell invasion [19]. Both ACH-3P and iOTR cells have low invasion; therefore, it would be hard to see a further reduction in invasion. Moreover, cell proliferation and invasion are two distinct and critical processes during placental development. Reduced trophoblast cell proliferation without reduced invasion can be sufficient to cause impaired conceptus elongation and attachment. These results suggest that the LIN28-*let-7* miRNA axis plays a role in proliferation of immortalized trophoblast cells by regulating the expression of genes associated with cell proliferation. We suggest that the iOTR cells overexpressing LIN28A and LIN28B would be a better choice to study molecular mechanisms in ovine trophoblast cells compared to iOTR cells with depleted LIN28A and LIN28B.

To our knowledge, this is the first in vivo study defining the role of LIN28-*let*-7 miRNA axis in early placental development by trophoblast-specific RNAi of LIN28A or LIN28B. Due to a wide range of *let-7* miRNAs target genes, as well as the ability of LIN28 to directly bind the mRNA of different genes, LIN28 knockdown might be affecting many different genetic pathways in trophoblast cells, which are yet to be explored. Knockdown of LIN28A or LIN28B and high *let-7* miRNAs in TE led to reduced conceptus elongation, which can result in impaired placentation, fetal growth restriction, loss of pregnancy, and reduced fertility in domestic ruminants. We recently showed that term human placentas from IUGR pregnancies have low LIN28 and high *let-7* miRNAs [18]. MicroRNAs can be easily measured in tissue biopsies, blood, and other biological samples. Based on our studies, we suggest that low LIN28 or high *let-7* miRNAs in placenta could be detected in blood and be used as potential biomarkers for intrauterine growth restriction.

## 4. Materials and Methods

### 4.1. Lentivirus Vector Construction for shRNA Expression

Lentiviral infection was used to stably integrate and express shRNA targeting LIN28A or LIN28B mRNA in the host cell. Lentiviral vectors were constructed using the protocol previously described by Baker et al. [61]. Briefly, LIN28A targeting shRNA, LIN28B targeting shRNA, or scrambled control shRNA sequence (Table S1) were first cloned into the pLKO.1 vector (plasmid 10878, Addgene, Cambridge, MA, USA), which contained the human U6 promoter upstream of cloning site for shRNA cassettes. The human U6 promoter and downstream LIN28A/LIN28B/SC shRNA sequence within pLKO.1 was PCR amplified using a forward primer with a 5′ XbaI restriction site(5′-TCTAGATTCACCGAGGGCCTATTTCCC-3′) and a reverse primer containing a 3′ XhoI restriction site (5′-GAATACTGCCATTTGTCTCGAGGTCG-3′). The resulting PCR amplicon was gel purified and cloned into the StrataClone PCR cloning vector using StrataClone PCR Cloning KIT (Agilent, Santa Clara, CA). The human U6 promoter and LIN28A/LIN28B/SC shRNA DNA fragment was digested from StrataClone PCR cloning vector using XbaI/XhoI restriction enzymes. Subsequently, the DNA fragment was ligated into the pLL3.7 vector also digested with XbaI/XhoI. Insertion of the human U6 promoter and LIN28A/LIN28B/SC shRNA sequence into pLL3.7 was verified by sanger sequencing.

### 4.2. Lentivirus Vector Construction for Overexpression of LIN28A and LIN28B

To overexpress LIN28A and LIN28B, pCDH lentiviral expression vector (System Biosciences, Palo Alto, CA, USA) was used. The mRNA was extracted from day 16 TE using RNeasy Mini Kit (Qiagen Inc. Germantown, MD, USA) following the manufacturer’s protocol, and then reverse transcribed to cDNA using iScript cDNA synthesis kit (Bio-Rad Laboratories, Hercules, CA, USA). The cDNA was amplified using PCR primers for LIN28A or LIN28B (Supplementary Table S2). The PCR primers included restriction sites for NheI and SwaI restriction enzymes. The resulting PCR amplicons were gel purified and cloned into the StrataClone PCR cloning vector using StrataClone PCR Cloning KIT (Agilent, Santa Clara, CA). StrataClone vector with successful cloning of PCR product was double digested using NheI/SwaI. The double digested product was cloned in double digested pCDH vector and confirmed by sanger sequencing.

### 4.3. Lentiviral Vector for Immortalizing Passaged Ovine Trophoblast Cells

To immortalize the OTR cells, pLV-hTERT-IRES-hygro was used (Addgene, Watertown, MA, USA, Plasmid # 85140) [67]. The pLV-hTERT-IRES-hygro vector-based lentiviral particles expressed human telomerase reverse transcriptase (hTERT) in the infected cells.

### 4.4. Production of Lentiviral Particles

To generate lentiviral particles, three vectors were used including transfer vector (LL3.7 or pCDH or pLV-hTERT-IRES-hygro), packaging plasmid (psPAX2 from Addgene, Watertown, MA, USA, Plasmid # 12260), and envelope plasmid (pMD2.G from Addgene, Watertown, MA, USA, Plasmid # 12259). The 293FT cells (Invitrogen, Carlsbad, CA, USA) were cultured in dulbecco’s modified eagle medium (DMEM) with high-glucose supplemented with 10% heat-inactivated fetal bovine serum (FBS) and 1x penicillin-streptomycin-amphotericin B (PSA) solution, at 37 °C and 5% CO_2_. Then, 8.82 µg transfer vector DNA, 6.66 μg psPAX2 packaging plasmid DNA, and 2.70 μg pMD2.G envelope plasmid DNA was mixed with 180 µL of polyfect transfection reagent (Qiagen Inc., Germantown, MD, USA) and the final volume was brought up to 855 µL using DMEM high-glucose media without any supplements. The plasmids-polyfect mixture was incubated at room temperature for 10 min and then gently mixed in the media on 70-80 % confluent 293FT cells. Cells were incubated for 4–6 h at 37 °C and 5% CO2. After incubation time, the transfection media was replaced by fresh DMEM high-glucose media supplemented with 10% FBS and 1x PSA solution. After 72 h, the medium containing lentiviral particles was collected and ultra-centrifuged over a 20% sucrose cushion at 25,000 RPM for 2 h at 4 °C. LL3.7 vector-based lentiviral particles were resuspended in chemically defined medium-2 (CDM-2), whereas pCDH or pLV-hTERT-IRES-hygro vector-based lentiviral particles were resuspended in 1x PBS, aliquoted, and stored at −80 °C.

To infect the cells by pCDH or pLV-hTERT-IRES-hygro based-lentiviral particle, the median tissue culture infectious dose (TCID50) of lentiviral particles was calculated. The frozen viral aliquot was resuspended in 0.5-1 mL of appropriate media with 8 μg/mL polybrene. The target cells were incubated with lentiviral particles at multiplicity of infection (MOI) of10 for 24 h at 37 °C and 5% CO_2_. After 72 h of culture, cells were selected with appropriate selection antibiotic. The LL3.7 vector-based lentiviral particles were tittered by infecting human embryonic kidney (HEK) cells and counting green fluorescent protein (GFP)-positive cells [61].

### 4.5. Blastocyst Collection and Transfer

All animal procedures were approved by Institutional Animal Care and Use Committee at Colorado State University, Fort Collins, Colorado, USA. Blastocysts collection and transfer was done following the procedure previously described by Baker at al [61]. A group of 12 ewes at day 6-12 of estrus were synchronized by two intramuscular injections of prostaglandin F-2α (PGF-2α) at 10 mg/dose given at interval of 4 h (Lutalyse, Pfizer, New York, NY). After 48 h of estrus synchronization, 4 ewes were separated to be used as recipients while 8 donor ewes were bred by intact rams. At day 9, donor ewes were euthanized using pentobarbital sodium (90 mg/kg IV, Pentasol, Vibrac, Fort Worth, TX, USA), and blastocysts were flushed from the uterus using DMEM-F-12 (1:1) medium supplemented with 0.25% BSA. The hatched blastocysts were infected with 100,000 shRNA expressing lentiviral particles in a 100 µL drop of CDM-2 media with 5 µg/mL polybrene (Sigma-Aldrich, St. Louis, MO, USA). Blastocysts with lentiviral particles were kept in incubator for 4–5 h at 5% CO2, 5% O2, and 38.5 °C. Overnight-fasted recipient ewes were sedated using ketamine (12.5 mg/kg IV, Ketacine, VetOne, Boise, ID, USA) and diazepam (0.125 mg/kg IV, Hospira, Lake Forest, IL, USA). Surgical procedure was performed under general anesthesia on 2 L/min O2 and 2–4% isoflurane (Fluriso, VetOne, Boise, IS, USA). A total of 23 blastocysts were transferred including 6 SC, 7 AKD, and 10 BKD. One blastocyst was transferred in each recipient.

### 4.6. Tissue Collection

For analysis of LIN28A or LIN28B knockdown and its effect on conceptus elongation, terminal surgeries were conducted on recipient ewes at 16 days gestational age (dGA), and tissues were collected. Conceptuses were flushed from the uterus using DMEM-F-12 (1:1) medium. After separating the embryo, trophectoderm length was measured, and both embryo and TE were snap frozen. TE samples were used to extract mRNA, miRNA, or proteins for further analysis.

### 4.7. Cell Lines

Day 16 TE from 3 non-infected pregnancies was minced in DMEM-F-12 (1:1) medium supplemented with 10% bovine serum albumin, 1x penicillin-streptomycin-amphotericin B solution, 10 µg/mL insulin, 0.1 mM non-essential amino acids, 2 mM glutamine, and 1 mM sodium pyruvate. The minced tissue was spun down at 1000 rpm for 5 min and the supernatant was incubated in a 100-mm collagen-treated tissue culture dish at 37 °C and 5% CO_2_. After 24 h, the cells attached to the plate were washed and incubated with fresh complete medium. After 48–72 h, the cells were passaged and later collected at passage number 4–6 at 70–80% confluency to extract mRNA, miRNA, and proteins for further analysis. Western blot analysis for cytokeratin-7 (CK-7) was done to confirm the phenotype of OTR cells. To generate immortalized ovine trophoblast cells (iOTR cells), the OTR cells were infected with pLV-hTERT-IRES-hygro based lentiviral particles resuspended in complete DMEM-F12 (1:1) medium supplemented with 8 µg/mL polybrene transfection reagent. The media with viral particles was replaced with fresh media after 24 h. The cells were selected in complete DMEM-F12 (1:1) medium supplemented with 300–500 µg/mL hygromycin B (Sigma-Aldrich, St. Louis, MO, USA).

### 4.8. Overexpression of LIN28A and LIN28B

To overexpress *LIN28* genes in iOTR cells, pCDH-LIN28A or pCDH-LIN28B-based lentiviral particles were used to infect iOTR cells at 70–80% confluency in one well of a 12-well plate (Corning Inc., Corning, NY, USA). After 48–72 h, the infected cells were selected using 2–4 µg/mL puromycin. Successful gene knockin was confirmed using real-time PCR and Western blot analysis. We generated iOTR cells with knockin of LIN28A (AKI) or LIN28B (BKI) and iOTR cells infected with empty-expression vector-based lentiviral particles to use as expression vector control (EVC).

### 4.9. RNA Extraction and Real-Time PCR

For real-time PCR analysis, mRNA was isolated from day 16 sheep TE, OTR cells, and iOTR cells using RNeasy Mini Kit (Qiagen Inc. Germantown, MD, USA), following the manufacturer’s protocol. The mRNA was reverse transcribed to cDNA using iScript cDNA synthesis kit (Bio-Rad Laboratories, Hercules, CA, USA). Real-time PCR reactions were run in triplicate in 384-well plates, using 10 µL reaction volume in each well. The reaction volume included 5 µL of 2x Light-Cycler 480 SYBR Green I Master (Roche Applied Science, Penzberg, Germany), 50 ng reverse-transcribed mRNA, and 1 µM of target-specific forward and reverse primers. Primer sequences used for real-time PCR are listed in Supplementary Table S2. PCR reactions were incubated in the Light-Cycler 480 PCR machine (Roche Applied Science, Penzberg, Germany) at the following cycling conditions: 95 °C for 10 min, 45 cycles of 95 °C for 30 s, 55 °C for 1 min, and 72 °C for 1 min. Relative mRNA levels were normalized using *RPS15.* For miRNA profiling, total RNA was extracted using a miRNeasy Mini Kit (Qiagen Inc. Germantown, MD, USA), following the manufacturer’s protocol. Then, 300 ng total RNA was reverse-transcribed to cDNA using miScript RT II kit (Qiagen Inc. Germantown, MD, USA). Real-time PCR reactions were run in triplicate in 384-well plates, using 10 µL reaction volume in each well. The reaction volume included 5 µL of 2x QuantiTech SYBR Green Master Mix (Qiagen Inc. Germantown, MD, USA), 3 ng cDNA, 1x miScript universal primer (Qiagen Inc. Germantown, MD, USA), and 1x miScript assay for *let-7* miRNAs (*let-7a, let-7b, let-7c, let-7d, let-7e, let-7f, let-7g, let-7i*). These reactions were incubated in the Light-Cycler 480 PCR machine (Roche Applied Science, Penzberg, Germany) at the following cycling conditions: 95 °C for 15 min, 45 cycles of 94 °C for 15 s, 55 °C for 30 s, and 70 °C for 30 s. Relative miRNA levels were normalized using *SNORD-48*.

### 4.10. Protein Extraction and Western Blot

Western blot analysis was performed using whole cell lysate to quantify proteins in cells and tissue samples. For protein extraction, cell pellets were resuspended in 200–400 µL radioimmunoprecipitation assay (RIPA) buffer (20 mM Tris, 137 mM NaCl, 10% glycerol, 1% nonidet *p*-40, 3.5 mM sodium dodecyl sulfate (SDS), 1.2 mM sodium deoxycholate, 1.6 mM ethylenediaminetetraacetic acid (EDTA), pH 8) containing 1x protease/phosphate inhibitor cocktail (Sigma-Aldrich, St. Louis, MO, USA). Whole cell lysate was incubated on ice for 5 min and then centrifuged at 14,000 g for 5 min to remove cell debris. To extract protein from day 16 TE, the tissue was homogenized in RIPA buffer. Homogenized samples were sonicated using a Bioruptor Sonication System (Diagenode, Denville, NJ, USA) for 5 cycles of 30 s “ON” and 30 s “OFF”. Sonicated samples were centrifuged at 14,000 g for 5 min to remove debris. Protein concentration was measured using the bicinchoninic acid (BCA) protein assay kit (ThermoFisher, Waltham, MA, USA). Protein was separated in 4–15% Bis-Tris gels (Bio-Rad Laboratories, Hercules, CA, USA) at 90 volts for 15 min and 125 volts for 60 min, and then transferred to 0.45 μm pore size nitrocellulose membrane (Bio-Rad Laboratories, Hercules, CA, USA) at 100 volts for 2 h at 4 °C. The membranes were then blocked in 5% non-fat dry milk solution in tris buffered saline with tween 20 (TBST) (50 mM Tris, 150 mM NaCl, 0.05% Tween 20, pH 7.6) for 1 h at room temperature. After blocking, the membranes were washed 3 times with 1x TBST for 5 min each, and then incubated at 4 °C overnight with specific primary antibody. After overnight incubation, the membranes were washed 3 times with 1x TBST for 5 min each. After washing, the membranes were incubated with appropriate secondary antibody conjugated to horseradish peroxidase for 1 h at room temperature. After removing the secondary antibody, the membranes were washed following the same procedure and developed using Super Signal WestDura Extended Duration Substrate (ThermoFisher, Waltham, MA, USA) and imaged using ChemiDoc XRS+ chemiluminescence system (Bio-Rad Laboratories, Hercules, CA, USA). The images were quantified using Image-Lab software (Bio-Rad Laboratories, Hercules, CA, USA). To normalize protein quantity, β-actin, α-tubulin, or glyceraldehyde 3-phosphate dehydrogenase (GAPDH), was used as loading control. Each experiment was repeated on three replicates. The antibodies used and their dilutions are listed in Table S3.

### 4.11. Cell Proliferation Assay

Cell proliferation was measured using Quick Cell Proliferation Assay Kit (Abcam, Cambridge, MA, USA) following manufacturer’s protocol. This assay is based on cleavage of tetrazolium salt (WST-1) to formazan by mitochondrial dehydrogenases. EVC, AKI, and BKI iOTR cells were plated to a density of 1000 cells/100 µL in 96-well tissue culture plates, with four replicates of each cell type. After 4, 24, 48, or 96 h of plating the cells, 10 µL WST-1 reagent was added in each well followed by incubation for 2 h in standard culture conditions. Absorbance was measured using Cytation 3 Multi-Mode Reader (BioTek Instruments, Inc., VT, USA) at 440 nm with reference wavelength of 650 nm.

### 4.12. Matrigel Invasion Assay

Cell invasion was measured using Corning BioCoat Tumor Invasion System (Corning, New York, NY, USA) following manufacturer’s protocol. EVC, AKI, and BKI iOTR cells were stained with CellTracker™ Green 5-chloromethylfluorescein diacetate (CMFDA) (Invitrogen, Carlsbad, CA, USA). Four replicates of each cell line were plated at a density of 10,000 cells/500 µL DMEM/F-12 (1:1) media without phenol red and fetal bovine serum, in a 24-multiwell insert plate with 8 µm pore size polyethylene terephthalate membrane coated with uniform layer of matrigel matrix. DMEM/F-12 (1:1) media without any cells was added in four wells to be used as blank. In the bottom wells, 750 µL of DMEM/F-12 (1:1) media with 10% fetal bovine serum was added. Plates were read at 2, 4, 24, and 48 h after plating the cells using Cytation 3 Multi-Mode Reader (BioTek Instruments, Inc., VT, USA) with top and bottom reading ability, at 492 nm excitation and 517 nm emission wavelengths. The invasion index was calculated based on relative fluorescent units (RFU) using the formula: (RFU of cells at the bottom/RFU of cells at top + RFU of cells at bottom) × 100.

### 4.13. Statistics

All data were analyzed using GraphPad Prism 7 Software. To determine significance of mRNAs, miRNAs, and proteins, t-test was used when comparing two groups and analysis of variance followed by Tukey’s honestly significant difference (HSD) post hoc test was done when comparing three groups. The *p* values less than 0.05 were considered statistically significant. The error bars in the figures indicate standard error of the mean (SEM).

## Supplementary Materials

Supplementary materials can be found at www.mdpi.com/xxx/s1.

## Abbreviations

OTR	non-immortalized ovine trophoblast cells
iOTR	immortalized ovine trophoblast cells
TE	trophectoderm
SC	scramble control shRNA
AKD	knockdown of LIN28A
BKD	knockdown of LIN28B
EVC	iOTR cells infected with empty lentiviral expression vectors
AKI	iOTR cells overexpressing LIN28A
BKI	iOTR cells overexpressing LIN28B
