# Supplementary material for: Trophectoderm-Specific Knockdown of LIN28 Decreases Expression of Genes Necessary for Cell Proliferation and Reduces Elongation of Sheep Conceptus
Source: Int J Mol Sci. 2020 Apr 6;21(7):2549. doi: 10.3390/ijms21072549 (PMC7177537; doi:10.3390/ijms21072549)
Supplement: Supplementary file 1 [file ijms-21-02549-s001.pdf]

Supplementary Materials

Supplementary table 1: shRNA oligos

LIN28A	Forward Oligo	5′ CCGGGCATCTGTAAGTGGTTCAACGTTCAAGAGACG TTGAACCACTTACAGATGCTTTTTG 3′
	Reverse Oligo	5′ AATTCAAAAAGCATCTGTAAGTGGTTCAACGTCTCT TGAACGTTGAACCACTTACAGATGC 3′
LIN28B	Forward Oligo	5′ CCGGGGATTCATCTCCATGATAAGCTTCAAGAGAGC TTATCATGGAGATGAATCCTTTTTG 3′
	Reverse Oligo	5′ AATTCAAAAAGGATTCATCTCCATGATAAGCTCTCT TGAAGCTTATCATGGAGATGAATCC 3′
SC	Forward Oligo	5′ CCGGAGTTAAAGGTTCGGCACGAATTCAAGAGATTC GTGCCGAACCTTTAACTCTTTTTG 3′
	Reverse Oligo	5′ AATTCAAAAAGAGTTAAAGGTTCGGCACGAATCTC TTGAATTCGTGCCGAACCTTTAACT 3′

Supplementary table 2: Primers for LIN28A and LIN28B Expression

LIN28A Expression	Forward Oligo	5′ GCTAGCCAGACTACCATGGGCTCTGTG 3′
	Reverse Oligo	5′ ATTTAAATACCCACTGTGGCTTCAATTC 3′
LIN28B Expression	Forward Oligo	5′ GCTAGCGCCGGAAAGAATTAGTTTCGC 3′
	Reverse Oligo	5′ ATTTAAATACATAACACATGACACCCT 3′

Supplementary table 3: Realtime RTPCR Primers

LIN28A	Forward Oligo	5′ GACAGGTGCTACAACTGTGGAG 3′
	Reverse Oligo	5′ ATGGCAGAGCTATGGATCTCTT 3′
LIN28B	Forward Oligo	5′ GCCTTGAATCAATACGGGTAAC 3′
	Reverse Oligo	5′ CTTCTTTGGCTGAGGAGGTAGA 3′
IGF2BP1 (IMP1)	Forward Oligo	5′ GCAACCTGAAGAAGGTGGAG 3′
	Reverse Oligo	5′ GCAGCCACGTCATTCTCATA 3′
IGF2BP2 (IMP2)	Forward Oligo	5′ TCCCGGGTAGACATCCATAG 3′
	Reverse Oligo	5′ GTGGGCCAAGATCTTCAGAG 3′

IGF2BP3 (IMP3)	Forward Oligo	5′ TGCCGCTGAGAAGTCAATTA 3′
	Reverse Oligo	5′ TCCGTCCTTCCTTACCAATG 3′
HMGA1	Forward Oligo	5′ AAGGGGAGCAAAAACAAGG 3′
	Reverse Oligo	5′ CCTCCTCTTCCTCCTTCTCC 3′
ARID3B	Forward Oligo	5′ AAGGTGATGGAGTCCCAGTG 3′
	Reverse Oligo	5′ CCTCTTCTGACAGCCTGGAC 3′
c-MYC	Forward Oligo	5′ CTTCTCCCCCTCCTCTGACT 3′
	Reverse Oligo	5′ GCCTCTTTTCCACAGAGACAA 3′
S15	Forward Oligo	5′ ATCATTCTGCCCGAGATGGTG 3′
	Reverse Oligo	5′ TGCTTGACGGGCTTGTAGGTG 3′

Table 4: Antibodies

Protein	Vendor	Catalog #	Host Species	Dilution used	Band size
LIN28A	Abcam	ab63740	Rabbit	1:1000	30 kDa
LIN28B	Bethyl Labs	A303-588A	Rabbit	1:2000	35 kDa
IGF2BP1	Abcam	ab82968	Rabbit	1:500	63 kDa
IGF2BP2	Abcam	ab124930	Rabbit	1:3000	60-66 kDa
IGF2BP3	Abcam	ab225697	Rabbit	1:3000	64 kDa
c-MYC	Abcam	ab32	Mouse	1:1000	45 kDa
HMGA1	Abcam	ab226850	Rabbit	1:1000	17 kDa
ARID3B	Bethyl Labs	A302-565A	Rabbit	1:4000	70 kDa
GAPDH	Abcam	ab9485	Rabbit	1:3000	37 kDa
β-actin	SCBT	sc-47778	Mouse	1:2000	45 kDa
α-tubulin	Abcam	ab4074	Rabbit	1:3000	50 kDa
Goat anti-rabbit (HRP)	Abcam	ab97051	Goat	1:3000	Secondary (HRP)
Goat anti-mouse (HRP)	Abcam	ab97023	Goat	1:3000	Secondary (HRP)
